# Stronger wind, smaller tree: Testing tree growth plasticity through a modeling approach

**DOI:** 10.3389/fpls.2022.971690

**Published:** 2022-11-10

**Authors:** Haoyu Wang, Jing Hua, Mengzhen Kang, Xiujuan Wang, Xing-Rong Fan, Thierry Fourcaud, Philippe de Reffye

**Affiliations:** ^1^ The State Key Laboratory of Management and Control for Complex Systems, Institute of Automation, Chinese Academy of Sciences, Beijing, China; ^2^ Beijing Engineering Research Center of Intelligent Systems and Technology, Institute of Automation, Chinese Academy of Sciences, Beijing, China; ^3^ School of Artificial Intelligence, University of Chinese Academy of Sciences, Beijing, China; ^4^ Engineering Research Centre for Waste Oil Recovery Technology and Equipment, Ministry of Education, Chongqing Technology and Business University, Chongqing, China; ^5^ CIRAD, AMAP, Univ Montpellier, CIRAD, CNRS, INRAE, IRD, Montpellier, France

**Keywords:** functional-structural plant model, mechanical model, critical wind speed, tree breakage, optimization, thigmomorphogenesis

## Abstract

Plants exhibit plasticity in response to various external conditions, characterized by changes in physiological and morphological features. Although being non-negligible, compared to the other environmental factors, the effect of wind on plant growth is less extensively studied, either experimentally or computationally. This study aims to propose a modeling approach that can simulate the impact of wind on plant growth, which brings a biomechanical feedback to growth and biomass distribution into a functional–structural plant model (FSPM). Tree reaction to the wind is simulated based on the hypothesis that plants tend to fit in the environment best. This is interpreted as an optimization problem of finding the best growth-regulation sink parameter giving the maximal plant fitness (usually seed weight, but expressed as plant biomass and size). To test this hypothesis *in silico*, a functional–structural plant model, which simulates both the primary and secondary growth of stems, is coupled with a biomechanical model which computes forces, moments of forces, and breakage location in stems caused by both wind and self-weight increment during plant growth. The Non-dominated Sorting Genetic Algorithm II (NSGA-II) is adopted to maximize the multi-objective function (stem biomass and tree height) by determining the key parameter value controlling the biomass allocation to the secondary growth. The digital trees show considerable phenotypic plasticity under different wind speeds, whose behavior, as an emergent property, is in accordance with experimental results from works of literature: the height and leaf area of individual trees decreased with wind speed, and the diameter at the breast height (DBH) increased at low-speed wind but declined at higher-speed wind. Stronger wind results in a smaller tree. Such response of trees to the wind is realistically simulated, giving a deeper understanding of tree behavior. The result shows that the challenging task of modeling plant plasticity may be solved by optimizing the plant fitness function. Adding a biomechanical model enriches FSPMs and opens a wider application of plant models.

## 1 Introduction

Environmental conditions, such as light, temperature, and humidity, influence the physiological processes and structural development of trees. Wind, being almost ubiquitous in nature, affects tree growth, reproduction, and even survival (Moulia et al., 2006; de Langre, 2008; Lopez et al., 2011). The “compensating mechanism” whereby advantageous modifications of a tree’s phenotype can emerge to adapt to the wind conditions has already been discovered (Whitehead, 1962; Whitehead & Luti, 1962). The term thigmomorphogenesis has been coined, which refers to a phenomenon that trees demonstrate plasticity in reaction to the external mechanical stimulus (Jaffe, 1973; Jaffe & Forbes, 1993; Telewski & Pruyn, 1998). Considerable support to this hypothesis has been provided by experimental observations: when trees were exposed to wind forces or other biomechanical perturbations, total dry weight, in particular stem dry weight, decreased, and biomass allocation among organs altered (Bonnesoeur et al., 2016). A reduction in both stem height and leaf area, but an increase in diameter (Whitehead & Luti, 1962; Jaffe & Forbes, 1993; de Langre, 2008), has been observed. Allometry of axes, e.g., the ratio between length and radius, is also affected. Wind sways induce strain force on trees, and it is demonstrated that plants can sense strains they are subjected to (Coutand & Moulia, 2000; Coutand et al., 2014). In some circumstances, the secondary growth (increase in the stem diameter) is more sensitive to changes in environmental conditions than the primary growth (increase in the stem length) (Collet et al., 2001).

How does a plant adjust its behavior accordingly? A plant simulation system can be a helpful way to support or test biological hypotheses (Trewavas, 2017; Calvo et al., 2019). The functional-structure plant model (FSPM) is a key tool for biomechanical computation as it provides detailed information on plant structure and takes into account the interaction between plant architecture and physiological processes during plant growth (Fourcaud et al., 2008). Typically, FSPMs include two components: a structural model and a functional model. The one-directional or bidirectional interaction between these two sub-models is necessarily a feature of FSPMs (Perttunen et al., 1996; de Reffye et al., 2020). The bending of a tree stem has been under study when a very early coffee tree structure was simulated (de Reffye, 1976). The mechanics of orientation of a plant stem due to the changes in loading and stresses have been studied with biomechanical models (Fournier et al., 1994; Almeras et al., 2002; Taylor-Hell et al., 2005). Considering the dynamic growth of trees, an incremental biomechanical model has been introduced to calculate the deformation of a single growing stem (Fourcaud et al., 2003; Fourcaud & Lac, 2003), simulating the influence of wind and self-weight. The modeling work has been done at the scale of the stem (Coutand et al., 2011; Guillon et al., 2012) or even the whole plant (Fourcaud et al., 2003). However, the simulation of tree thigmomorphogenesis under a wind environment is hardly seen. The feedback between mechanics and plant growth has rarely been taken into account in growth models at the architectural scale (Eloy et al., 2017).

This study aims to explore tree growth plasticity under a wind environment in a mechanistic way through a modeling approach. To achieve this goal, firstly, a FSPM GreenLab (de Reffye et al., 2020) is coupled with a biomechanical model (Ancelin et al., 2004b) to simulate the effect of wind on biomechanical variables inside a full tree structure; secondly, a sensitivity analysis is performed to evaluate the effect of key parameters controlling the secondary growth; thirdly, tree plasticity is simulated through parameter optimization for different wind speeds searching the best parameter values that benefit tree growth. Thigmomorphogenesis is modeled as an emergent result of optimization in biomass reallocation.

The validation of this theoretical result can be made by experiments with different wind environments since parameters for secondary growth can be estimated inversely from measured data (Letort et al., 2008). However, as the sampling in such experiments is very costly, an *in silico* experiment remains an appealing tool to test hypotheses and inspire new thought. In a former study, the optimization of wood yield with the constraint of stability has been conducted (Qi et al., 2009), which also attempts to find the best parameter for secondary growth. The current work differs from it in several aspects: first, the wind environment is considered, while the former is limited to gravity. Secondly, this work couples the biomechanical and growth models, so that the incremental growth of trees is considered, while the former evaluates the mechanical properties of a straight adult tree. Thirdly, the biomechanical properties for all stems, including branches, are computed, while the previous work was limited to the main stem. The difference also lies in the constraint of the biomechanical stability of the tree.

## 2 Material and methods

### 2.1 System overview

**Figure 1:**
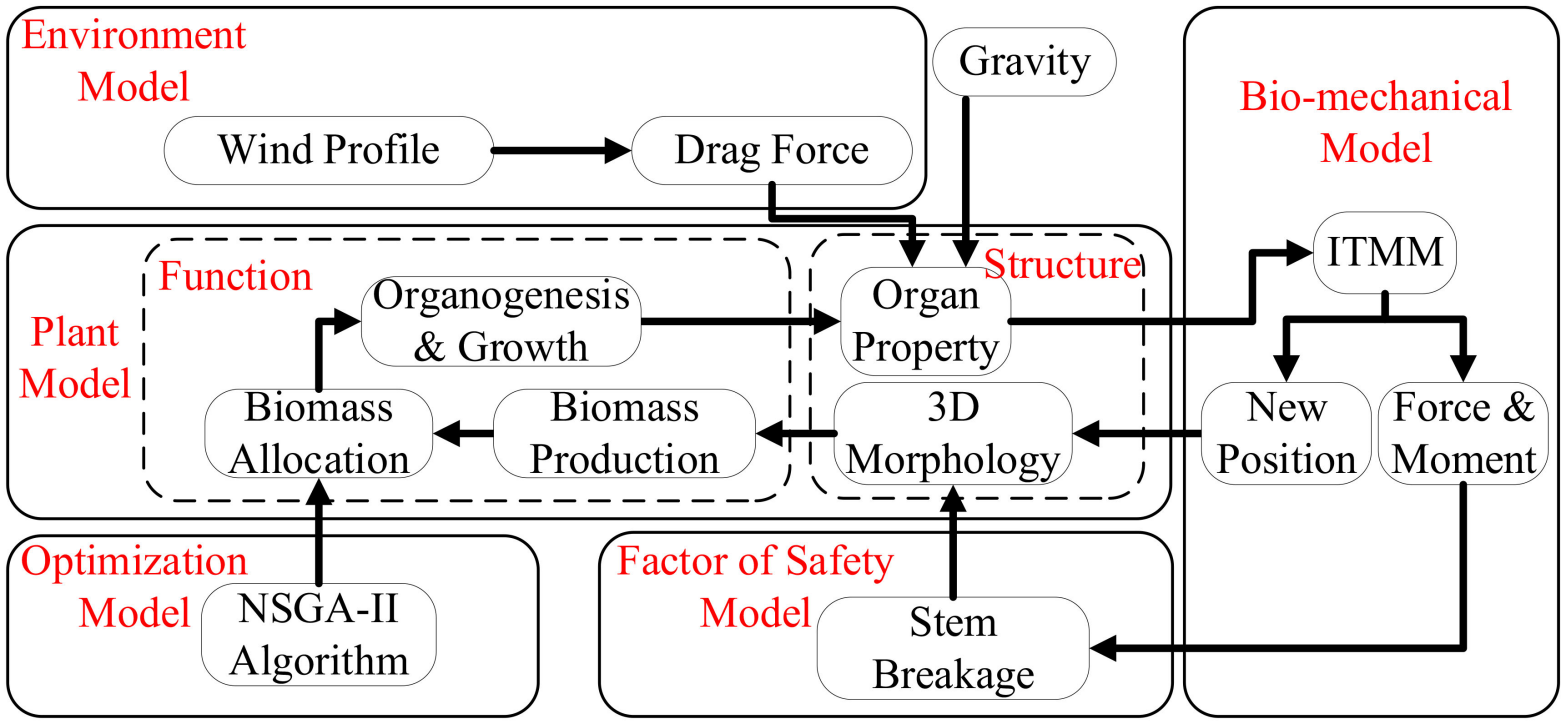
Schematic diagram of the modeling system.

The components of the whole system are shown in **Figure 1**. The plant model simulates step by step the tree structure and growth driven by a source–sink balance, giving the topological and geometrical tree structure with detailed information of the size and weight of individual organs (leaves, internodes, etc.). The wind environment model provides the wind profile and corresponding drag force. As this work is dedicated to wind, other environmental factors like light or temperature are not considered here, regarded as non-limiting. The biomechanical model computes the average force, moment, and new position of each internode under wind and gravity, for each growth step. The Factor of Safety Model checks whether the critical point of breakage is reached for every stem. The optimization model simulates plant plasticity by searching parameter values to maximize the objective function of tree growth while keeping the main stem unbroken.

**Figure 2:**
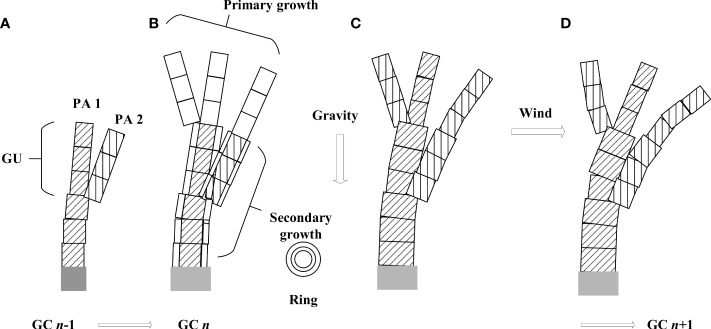
Illustration of tree development, primary and secondary growth, as well as the simulation of wind and gravity effect during a growth cycle. **(A)** plant top with two physiological ages (shown with different textures) at GC *n* - 1; **(B)** organogenesis, primary growth (blank rectangles) and secondary growth (new rings on existing rectangles) at GC *n*; **(C)** permanent deformation caused by increment in gravity; **(D)** temporary deformation caused by wind.

For a plant growth model, it is necessary to clarify at which level of detail the model is built. Here, the temporal scale corresponds to a growth cycle (GC), i.e., the time it takes to create a new growth unit (GU, **Figure 2A**), which is a year for temperate trees. The spatial scale corresponds to a phytomer, composed of an internode and its axillary leaves. A GU is composed of one or more phytomers of the same feature.

The integration of a dynamic plant growth process for plants and biomechanical model is as follows. At each GC, the organogenesis and organ growth take place, which creates new organs and increases the existing stem diameter (**Figure 2B**). A biomechanical model is called to compute force and moment as well as the new stem position caused by the increment in self-weight (**Figure 2C**). The deformation caused by self-weight is regarded as permanent. Given wind load, the wind-induced force leads to new destabilizations, and the biomechanical model is called again until a new equilibrium status, as well as the displacement of each internode, is reached (**Figure 2D**). However, after the withdrawal of wind, the plant recovers its original position as one can see in nature. This is achieved by running again the biomechanical module after the removal of wind force. This step is necessary as branch breakage induced by wind may have taken place, which influences self-weight distribution. In the next growth cycle, the plant development continues based on the deformed or broken structure of the last cycle.

### 2.2 Biomechanical model

Stem deformation is calculated at each internode *k* using the Incremental Transfer Matrix Method (ITMM) (Ancelin et al., 2004b), as in Eq. 1.


(1) $$\left\{ \begin{array}{l} D_{k+1}\;=\;T_{11}D_{k}+T_{12}(C_{k}-S_{k})+M_{1}F+M_{a}\\ S_{k+1}=T_{21}D_{k}+T_{22}(C_{k}+S_{k})+M_{2}F \end{array}\right.$$

where *D* is the displacement vector, which includes the positive translations of the cross-section center (m) and the positive rotations of the cross-section normal (radians) (see components in **Supplement A**), *S* is the stress and moment vector; *C* is the vector of the sum of all concentrated forces and moments at the branching node, with the incremental weight of the branch computed by the plant model; *F* is the vector of linear forces, caused by both the incremental self-weight and wind load (Eq. 9); and *T* and *M* are transformation matrices (see Eqs. S3-S6 and Eqs. S7-S12 in **Supplement A**). *M*_a_ denotes the maturation strains (MS) at the periphery of stems, associated with the formation of reaction wood (see Eq. S13 in **Supplement A**). The link between the biomechanical model and plant model lies in the concentrated forces and moments at the branching node (*C*), and the self-weight of internode (*F*), as the FSPM gives the 3D structure and the concentrated force of each sub-structure.

During the dynamic growth of plants, in each growth cycle, tree secondary growth takes place by adding a new layer to the existing stems. In the biomechanical model, a tree stem is thus regarded as a sequence of multilayer three-dimensional beam elements (Ancelin et al., 2004b). The biomechanical calculation consists of two steps. Firstly, compute the new incremental loads and the moments (caused by self-weight) on each internode. This is done using the idea of sub-structure (Kang et al., 2008), starting from the twigs of the highest branching order until the trunk (main stem). In ITMM, only the increment of weight must be considered in a growth step. Leaves, fruits, and flowers are considered as concentrated forces and moments for each internode. Dead or pruned branches are removed from the insertion point on the parent branch. Secondly, compute the new incremental displacement based on the last equilibrium, until the top of the main stem. Once this process is completed, the result is validated by examining the biomechanical boundary condition (BC) at the free top of the tree (Ancelin et al., 2004a), so that there is hardly any force or moment at each tip. If the BCs are not met, the last two steps are repeated.

When the wind is imposed, the displacement of the internode will take place, which changes the gravity center. With this increment of load, at each growth cycle, ITMM needs to be run multiple times to reach a new equilibrium, as if the plant swings before becoming stable. Similarly, when the wind calms down, iterated computation is called again. As a result, at each growth cycle, the equilibrium status of the tree with and without wind is updated. The iteration number can be set respectively for the main stem and branches.

### 2.3 Incremental weight in plant structure

The incremental weight of individual organs during plant growth is computed using the GreenLab model. GreenLab is a generic FSPM that has been applied for beech tree (*Fagus sylvatica* L.) (Letort et al., 2008), poplar tree (*Salicaceae*) (Yang et al., 2011), and Chinese pine (*Pinus tabulaeformis* Carr.) (Guo et al., 2012). For trees, a model with a finer temporal scale with stochastic behavior has been proposed (Kang et al., 2018), but here a deterministic model based on the growth cycle is adopted, where internodes created during the same GU have the same size. We recall here mainly components for secondary growth. See **Supplement B** for a more complete presentation, especially the primary growth.

For biomechanical computation, the output needed from GreenLab include the length and diameter of individual internodes; the weight, position, and gravity center of the aggregated branch; and the initial geometrical shape of the tree structure for computing the gravity center. The last is computed from the topological structure, the size of organs, and the insertion and phyllotaxy angle, which is not detailed here. The poplar (*Populus*) tree is selected for this study. Parameter settings used in this study are shown in **Table 1**.

**Table 1:** Parameter values of poplar tree.

Parameter	Definition	Value
*S*_p_	Total ground projection area available of the crown of plant; see Eq. S15 in **Supplement B**.	12 m^2^
*P*_m_	Maximum physiological age (PA); see Eq. 2.	4
*e*	Specific leaf weight; see Eq. S23 in **Supplement B**.	0.023 g·cm^-2^ (Yang et al., 2011)
$P_{p}^{b}$	Sink strength of leaf blade of PA *p*; see Eq. S16 in **Supplement B**.	1, 0.66, 0.32, 0.2, from PA 1 to PA *P*_m_ (Yang et al., 2011)
$P_{p}^{i}$	Sink strength of internode pith of PA *p*; see Eq. S16 in **Supplement B**.	0.875, 0.5, 0.3, 0.1, from PA 1 to PA *P*_m_
$P_{p}^{r}$	Relative sink strength of layer with PA *p*; see Eq. S22 in **Supplement B**.	0.8, 0.05, 0.05, 0.05
*α*	Internode allometry parameter; see Eq. S19 in **Supplement B**.	-0.28, -0.36, -0.26, -0.06 from PA 1 to PA *P_m_* (Yang et al., 2011)
β	Internode allometry parameter; see Eq. S19 in **Supplement B**.	6.51, 4.22, 3.77, 5.20 from PA 1 to PA *P_m_* (Yang et al., 2011)
$\rho_{p}^{pith}$	Density of pith; see Eq. S18 in **Supplement B**.	0.36 g·cm^-3^ (Kord et al., 2010)
$\rho_{p}^{layer}$	Density of layer; see Eq. 7	0.36-0.48 g·cm^-3^ (Kord et al., 2010)
MOR	Modulus of rupture	45 MPa (Grotta et al., 2005b)

#### 2.3.1 Weight of organs and substructure

The plant growth is based on source–sink regulation. Based on the pipe model, the demand for the secondary growth ( $D_{\text{sec}}^{\text{layer}}$ ) is proportional to the number of leaves in the last cycle $N_{p}^{b}(n-1)$ , using Eq. 2:


(2) $$D_{\text{sec}}^{\text{layer}}(n)=S_{\text{layer}}\sum_{p=1}^{P_{m}}N_{p}^{b}(n-1)$$

where *S*_layer_ denotes the sink strength of a new layer contributed by an individual leaf. It controls the proportion of produced biomass allocated to the secondary growth at each GC. Subscript *p* is the physiological age (PA), from 1 to maximum physiological age *P_m_*. Superscript *b* denotes the leaf blade. Symbol *n* denotes growth cycle *n*. In GreenLab, new rings of wood formed around the stem periphery. Each ring on a stem is contributed by numerous layers. Rings are modeled in two steps. Firstly, calculate the amount of biomass allocated to the secondary growth at GC *n* at the whole-plant scale, denoted by $Q_{\text{sec}}^{\text{layer}}(n)$ , as in Eq. 3:


(3) $$Q_{\text{sec}}^{\text{layer}}(n)=D_{\text{sec}}^{\text{layer}}(n)\frac{Q(n)}{D(n)}$$

Secondly, $Q_{\text{sec}}^{\text{layer}}(n)$ is allocated to each internode with two possible modes. Let parameter *λ* describe the way of biomass allocation associated with the secondary growth for each phytomer; the incremental biomass at GC *n* for an internode of PA *p* that showed GC *j* is described in Eq. 4:


(4) $$q_{\text{sec},p}^{\text{layer}}(j,n)=\left(\frac{1-\lambda }{D_{1}(n)}+\frac{\lambda \cdot s_{p}^{a}\left(j,n\right)}{D_{2}\left(n\right)}\right)\cdot l_{p}\left(j\right)\cdot P_{p}^{\text{r}}\cdot Q_{\text{sec}}^{\text{layer}}(n)$$

See Eq. S22 for demand for primary growth *D*_1_(*n*) and secondary growth *D*_2_(*n*), as well as other parameters. If *λ* = 0, it means that at each cycle the biomass for the layer is uniformly allocated to internodes of the same PA; tapering can appear because older internodes have more layers. If *λ* = 1, the allocation is also proportional to the total number of living leaves above the internode; since upper internodes have fewer leaves above, thus tapering is more obvious compared to the case of λ = 0. The weight of an individual internode that is born in cycle *j* is thus the result of primary and secondary growth:


(5) $$q_{p}^{i}(j,n)=q_{\text{pri},p}^{i}(j)+\sum_{i=j+1}^{n}q_{\sec,p}^{layer}(j,i)$$

Two key parameters that control the biomass allocation to the secondary growth are (1) parameter *S_layer_* controlling the amount of biomass allocated to the secondary growth at the whole-plant level (Eq. 2) and (2) parameter *λ* controlling the allocation of biomass for secondary growth among all internodes (Eq. 4) and, accordingly, stem shape, as shown in **Supplementary Figure S1**.

#### 2.3.2 Weight of plant and substructures

The weight of a branch (sub-structure) of PA *p* is the total weight of its bearing axis (stem) and all axillary axes (Kang et al., 2008). With the sub-structure method, the summing up starts from the branch of the highest PA (no sub-branch) until the main stem of PA 1. Similarly, the incremental weight of the branch can be obtained.

The total stem weight of the structure of the plant is the sum of the weights of the main stem and all branch stems, which is one of the object functions for optimization:


(6) $$Q_{\text{stem}}=\sum_{p=1}^{P_{m}}\sum_{j=1}^{n}N_{p}^{i}(j)q_{p}^{\text{i}}(j,n)$$

The number of internodes that are created at cycle *j*, , is updated in each cycle considering both organogenesis and possible stem breakage.

### 2.4 Size of organs

Considering that wood density is dependent on the radial position and height of the internode, parameter $\rho_{p}^{\text{layer}}(j,i)$ is set to describe the density of the layer in GC *i* for internode of PA *p* created at GC *j*. Then, at cycle *i*, the section area of layer added to such internodes is computed as


(7) $$s_{\text{sec},p}^{\text{layer}}(j,i)=\frac{q_{\text{sec},p}^{\text{layer}}(j,i)}{\rho_{p}^{\text{layer}}(j,i)*l_{p}(j)}$$

The radius of internode is finally the result of layer accumulation:


(8) $$r_{p}^{\text{i}}(j,n)=\sqrt{s_{p}^{\text{i}}(j,n)/\pi }=\sqrt{[s_{p}^{\text{pith}}(j)+\sum_{\text{i}=\text{j}+1}^{\text{n}}s_{\text{sec},p}^{\text{layer}}(j,i)]/\pi }$$

where *l_p_(j)* and $s_{p}^{\text{pith}}(j)$ are the length and section area of pith from primary growth (see **Supplement B**).

### 2.5Wind-induced force

According to literature (Mayhead, 1973; Niklas & Spatz, 2000; Ancelin et al., 2004a; Diener et al., 2010), the wind-induced drag force *F*w by wind on the *k*th element at the height *z* aboveground can be described as in Eq. 9:


(9) $$\begin{array}{l} F{\text{w}}_{k}(z)=\frac{1}{2}\rho_{air}C_{d}A_{k}u(z{)}^{2}\\ A_{k}=2l_{k}(z)r_{k}(z)\left|\sin\chi \right| \end{array}$$

where *ρ*_air_ is the air density (1.226 kg·m^-3^ when the air temperature is 15°C). *C*_d_ (dimensionless) is the drag coefficient, assumed to be 0.25 according to Koizumi et al. (2010). Differing from Sellier et al. (2008) where the exposed area is estimated from the biomass ratio, here *A_k_* (m^2^) is the frontal area of the *k*th internode, i.e., the projected area of the internode against the wind direction. It is computed with the length, diameter of the internode, and angle to the vertical direction of the internode, where *l* and *r* are the length and radius of the internode, respectively; *X* is the angle between this internode with the wind direction. *u(z)*is the wind speed at vertical height *z* which is given by a logarithmic wind profile.

Wind profile refers to the vertical distribution of wind speed. There are different wind profiles in the literature describing the horizontal mean wind speed according to the height aboveground (Niklas & Spatz, 2000). Some researchers (Irvine et al., 1997; Ancelin et al., 2004a) distinguished the wind profiles in forests located inside the stand and at the stand edge, including above and below the canopy. For trees standing in an open terrain, as assumed in this paper, a logarithmic wind profile is used (Ancelin et al., 2004b), which has provided the best fit for wind speeds empirically recorded within and just above the canopy (Niklas & Spatz, 2000).


(10) $$u(z)=\text{u}(h_{0})\frac{\text{ln}(z/z_{o})}{\text{ln}(h_{0}/z_{o})}$$

where *u*(*z*) (m·s^-1^) is the wind speed at vertical height *z*; *h*_0_ is the standard reference height of 10 m in Meteorology (Davenport, 1962); *z_0_* is the roughness length at stand edge (m). Ratio *z*_*o*_/*h*_0_ is set to 0.06 (Peltola & Kellomäki, 1993; Ancelin et al., 2004a).

### 2.6 Factor of safety

It is assumed that stem breakage takes place when its maximum longitudinal stress exceeds the Modulus of Rupture (MOR) of wood fibers (Jones, 1983; Gardiner et al., 2000). According to Jones (1983); Gardiner et al. (2000); Grotta et al. (2005), and Fjeld (2012), it is found that individual trees differed significantly in their MOR (range: 37–148 MN/m^2^). The maximum absolute value of longitudinal stress is found at the periphery of stem cross sections and depends on the bending moment (*M*, N·m ) of the stem and the third power of the stem diameter (Jones, 1983; Gardiner et al., 2000), as in Eq. 11:


(11) $${\text{Stress}}_{k}=\frac{32M_{k}}{\text{π}d_{k}^{3}}$$

Differing from Gardiner et al. (2000) who assumed that the stress is constant and thus calculated the stress only at breast height, here for each internode this stress is computed. The bending moment *M_k_* caused by the wind results from Eq. 1 (a component of *S_k_*). *d_k_* is the diameter of the *k*th stem internode as calculated in Eq. 8, and Stress*_k_* (Pa) is the longitudinal stress of internode *k.*

The critical wind speed (CWS) is defined as the minimum wind speed causing the breakage of the trunk, with the given parameter set. Higher CWS means that the tree is more wind-resistant.

The wind resistance can also be indicated by the top stem deflection of the trunk, defined as the angle between the trunk’s top and vertical direction.

### 2.7 Objective function

As mentioned above, the adaptation of trees to varying circumstances is modeled as an optimization problem. The optimization objective of the acclimatization is set to be the wood fresh weight *Q*_stem_ (total internode weight, Eq. 6) and the height of tree *H_tree_*. The reason for adding tree height as one of the objectives is that reaching a certain height is beneficial for light interception. Since in stronger winds the tree is more prone to breakage, thus it is expected that the tree’s reaction to the wind is to increase the radial growth. However, over-allocation to secondary growth will diminish the photosynthesis production and tree growth; thus, there is a balance between primary and secondary growth. The aim of optimization is to find the best balance. We choose the sink strength parameter for the secondary growth *S*_layer_ (Eq. 2) as the control variable.

Stem breakage under loads includes both trunk breakage and branch breakage (Lopez et al., 2011). Breakage on the trunk is likely to be fatal, while tree survival is often possible after branch breakage. Thus, here trunk breakage is considered a constraint condition.

In summary, this optimization problem with constraints is given by Eq. 12:


(12) $$\{\begin{array}{c} Max\;H_{\text{tree}}(S_{\text{layer}})\\ Max\;Q_{\text{stem}}(S_{\text{layer}})\\ subject\;to\;Stress_{\text{trunk}}<MOR \end{array}$$

The value of *MOR* is 45 MPa (**Table 1**). The multi-objective optimization algorithm of the Non-determined Sorting Genetic Algorithm II (NSGA-II) (Deb et al., 2000) from PAGMO (a C + + scientific library for massively parallel optimization) (Biscani & Izzo, 2020) is adopted. NSGA-II is one of the most popular multi-objective genetic algorithms. It has the advantages of fast-running speed and good convergence of the solution set, making it a benchmark of the performance of other multi-objective optimization algorithms. The population size is set as 100, the evolutionary algebra as 100, the crossover probability as 0.7, and the mutation probability as 0.4.

## 3 Results

### 3.1 The equilibrium of tree under wind load

**Figure 3:**
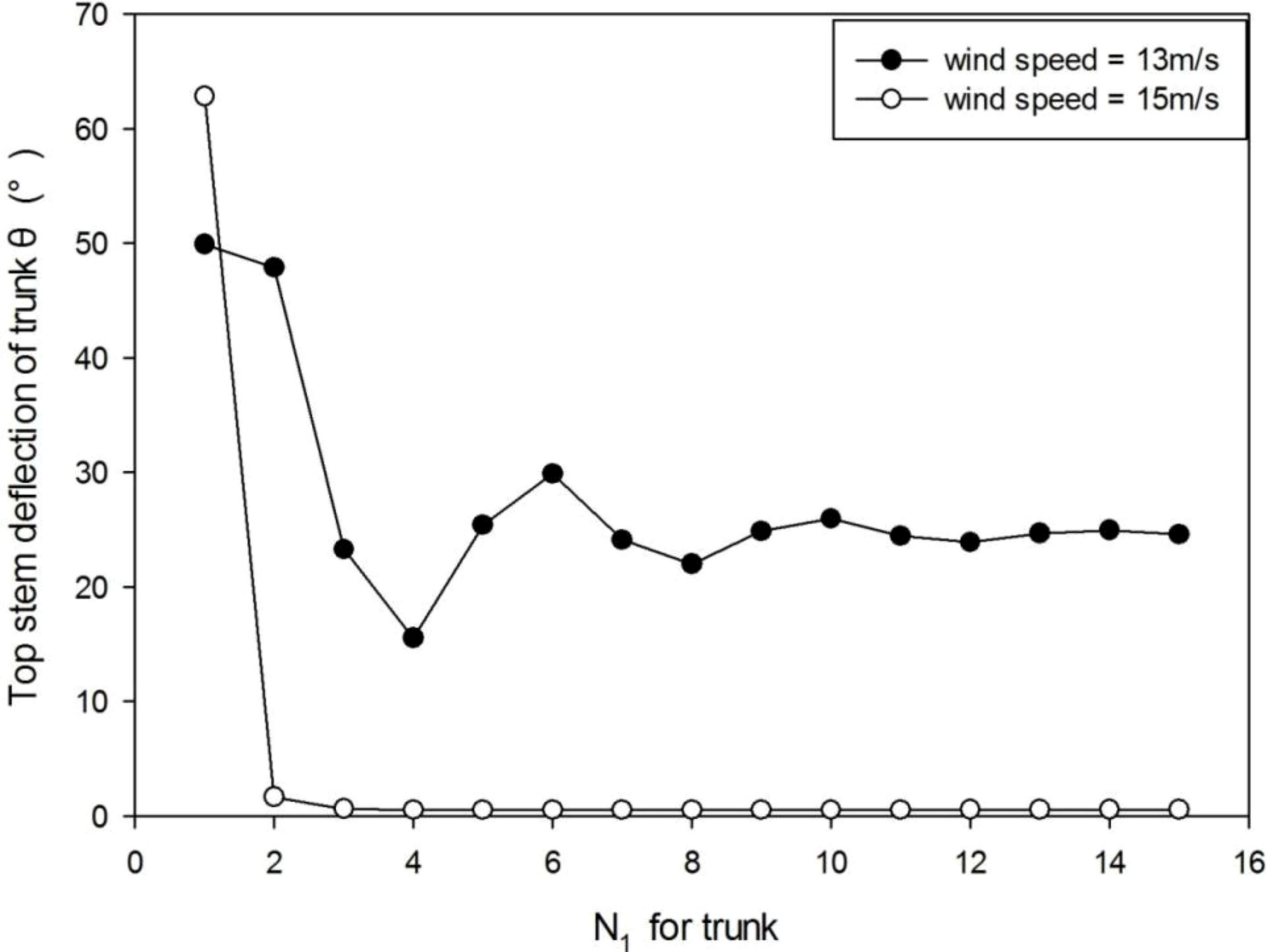
The stem deflection angle of the trunk under wind speeds of 13 m/s and 15 m/s. When wind speed is 13 m/s, it takes about 10 iterations before the stabilization. When wind speed is 15 m/s, trunk breakage takes place after the first iteration.

As mentioned above, the biomechanical calculation (Eq. 1) consists of two steps and requires multiple computational iterations to reach equilibrium. Under wind, the stem deformation stabilizes after several iterations or simply breaks under strong wind (**Figure 3**). At a wind speed of 13 m/s, the tree sways during about 10 iterations before the stabilization. When the wind speed is 15 m/s, trunk breakage takes place after the first iteration. With more iteration steps, the computational time increases linearly. With 10 iterations, at age 8 of the plant which means 8 years, it takes about 20 s for running the whole model in a PC of Core™ i7-8550U CPU 1.80 GHz, 16 GB RAM. Let *N_p_* be the iteration number for the substructure of PA *p*. According to the result, in the following computations, the iteration number of ITMM is set to 10 since the displacement error becomes negligible.

### 3.2 Numerical results

#### 3.2.1 Force and moment

**Figure 4:**
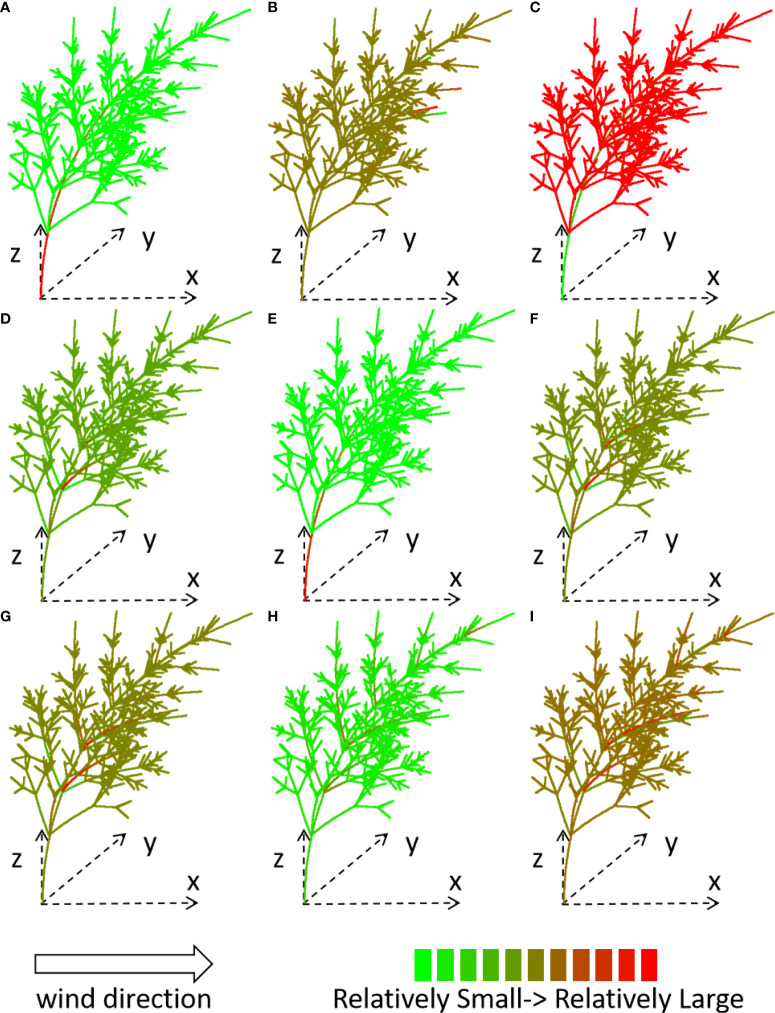
Computed force **(A-C)**, moment **(D-F)** and stress*_k._* **(G-I)** of *x-*, *y*-, *z*- component for the plant under wind speed 15 m/s, shown with different colors. *S*_layer_ = 4, *λ* = 0.1.

To visualize how the system works, the tree structure of GC 8 is simulated with parameter set *S*_layer_ = 4, *λ* = 0.1, under wind speed 15 m/s. The result is shown for the first iteration before trunk breakage. The displacement, force (**Figures 4A-C**, the first row), moment (**Figures 4D-F**, the second row), and stress*_k_* (**Figures 4G-I**, the third row) of each stem under a wind environment (which blows in the *x*-direction from left to right) are shown through tree shape and color, for the *x-*, *y*-, and *z*-components, respectively. The force is a vector. When the direction of the force is inconsistent with the direction of the coordinate axis, the force takes a negative value.

#### 3.2.2 Effect of wind speed

**Figure 5:**
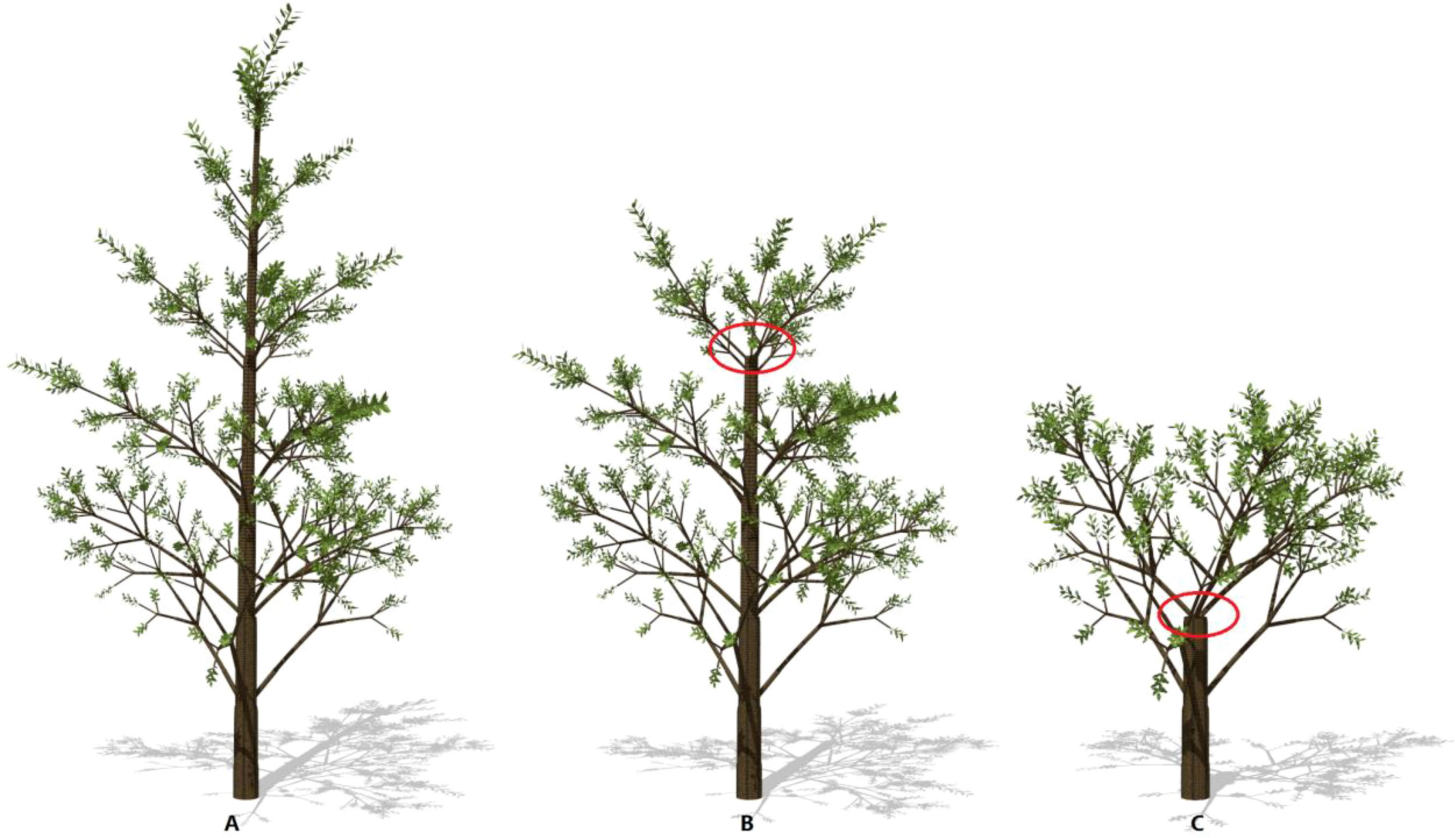
The effect of wind speed on tree breakage: 13m/s **(A)**, 15m/s **(B)**, 18m/s **(C)**, respectively. Result is shown under calm conditions. The remaining trunk biomass is 170.78, 159.85 and 120.58 kg, respectively. The circles indicate the points where the trunk breakage takes place. The plant age is 8, *S*_layer_ = 6, *λ* = 0.1.

To understand how wind speed can influence tree breakage, the tree shapes are shown in **Figure 5**. Here, *S*_layer_ and *λ* are set to 6 and 0.1, respectively. With increasing wind speed, more branches are lost because of breakage. The effect is evident on the top of the trunk.

### 3.3 Parameter sensitivity

#### 3.3.1 Parameter *S*_layer_

**Figure 6:**
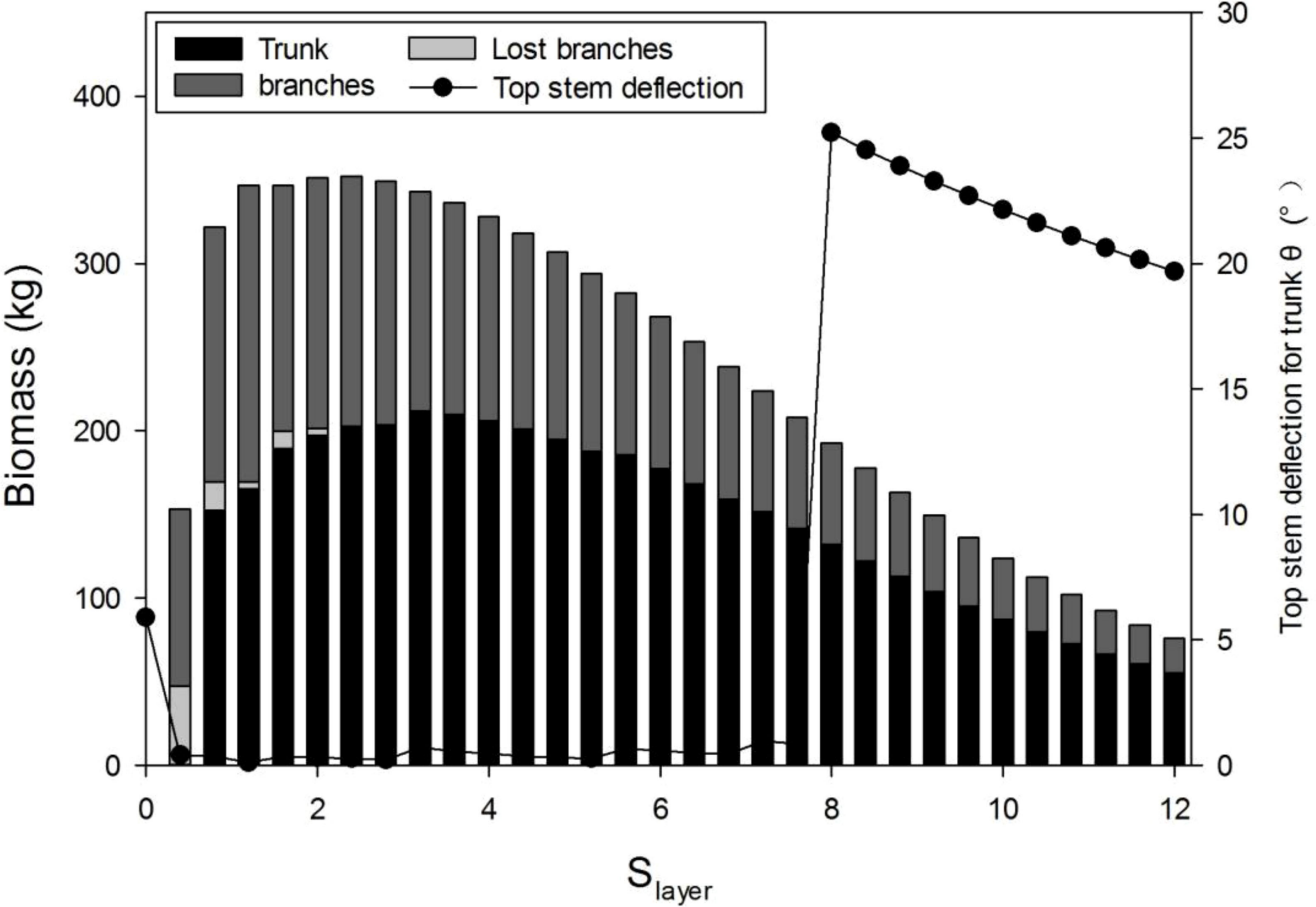
Influence of the sink strength for the secondary growth (*S*_layer_) on the wood biomass *Q*_stem_ and top step deflection for the trunk, under wind speed 15 m/s with *λ* = 0.4.

Before parameter optimization, it is useful to understand the model behavior using a sensitivity analysis. According to the GreenLab model, the biomass allocation to the secondary growth, regulated by parameter *S*_layer_ (Eqs. 2, 3), has twofold effects. On the one hand, the increase in biomass allocation to secondary growth increases the stem biomass. On the other hand, it can inhibit primary growth, thus decreasing the total leaf area and biomass production. In **Figure 6**, the stem biomass and top stem deflection for a trunk with different *S*_layer_ values are simulated. As expected, the peak value of the biomass of tree and trunk exists, which is independent of the wind. With a lower *S*_layer_ value, stem breakage is more evident; a sudden drop for *S*_layer_ *<*8 means trunk breakage. **Supplementary Figure S2** shows 3D tree shapes with different *S*_layer_ values, where broken branches are removed. With a higher *S*_layer_ value, the tree structure is more complete but smaller, because of the smaller leaf area for photosynthesis.

**Figure 7:**
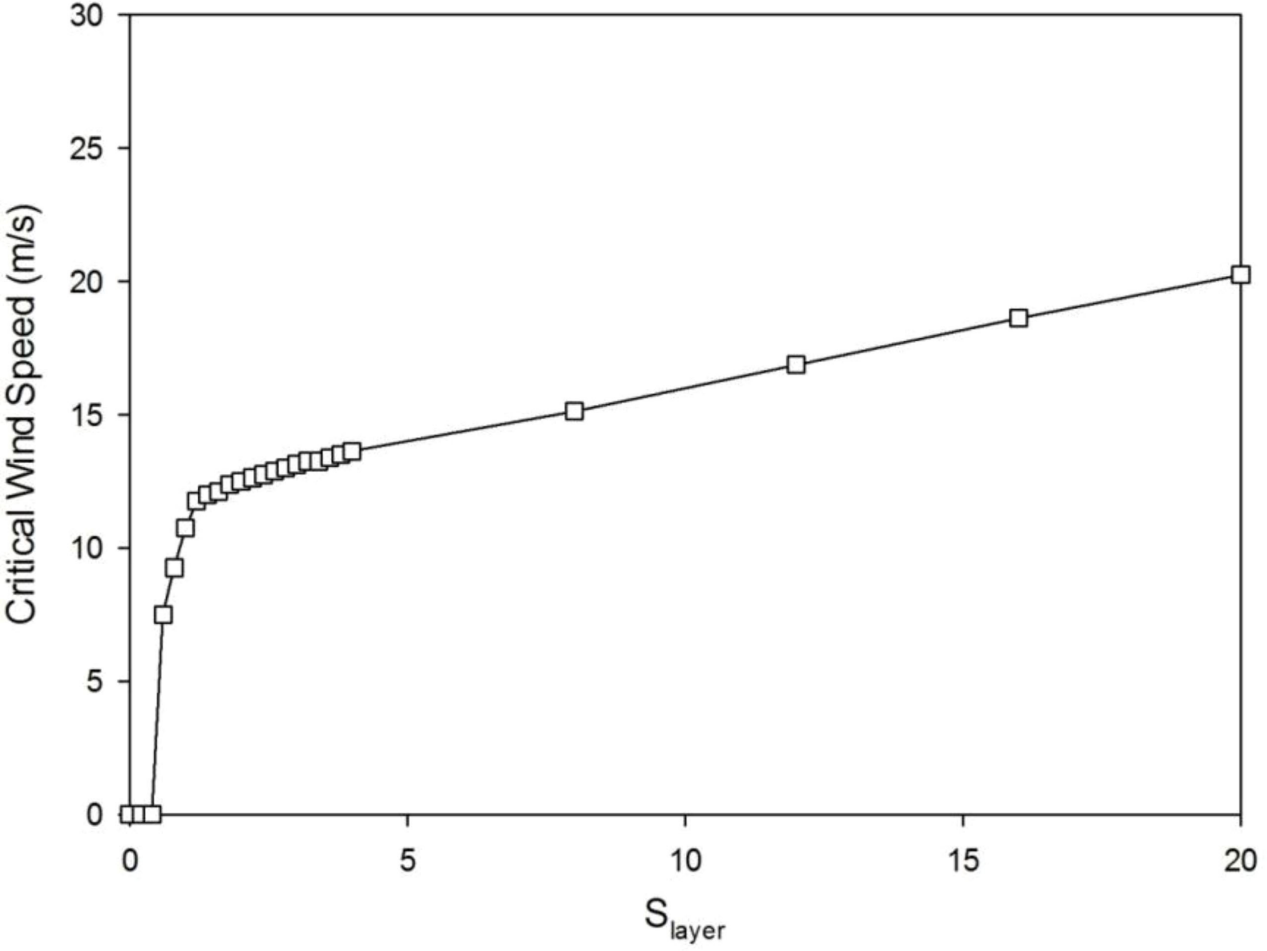
The critical wind speed (CWS) increases with *S*_layer_ values with *λ* = 0.4.

The critical value of wind speed (CWS) gradually increases with *S*_layer_ (**Figure 7**). It is because when more biomass was allocated to the secondary growth, the tree is easier to resist the breakage induced by wind. This shows from another point of view than the step deflection angle that with a bigger *S*_layer_, the tree is more resistant to wind.

#### 3.3.2 Parameter *λ*

**Figure 8:**
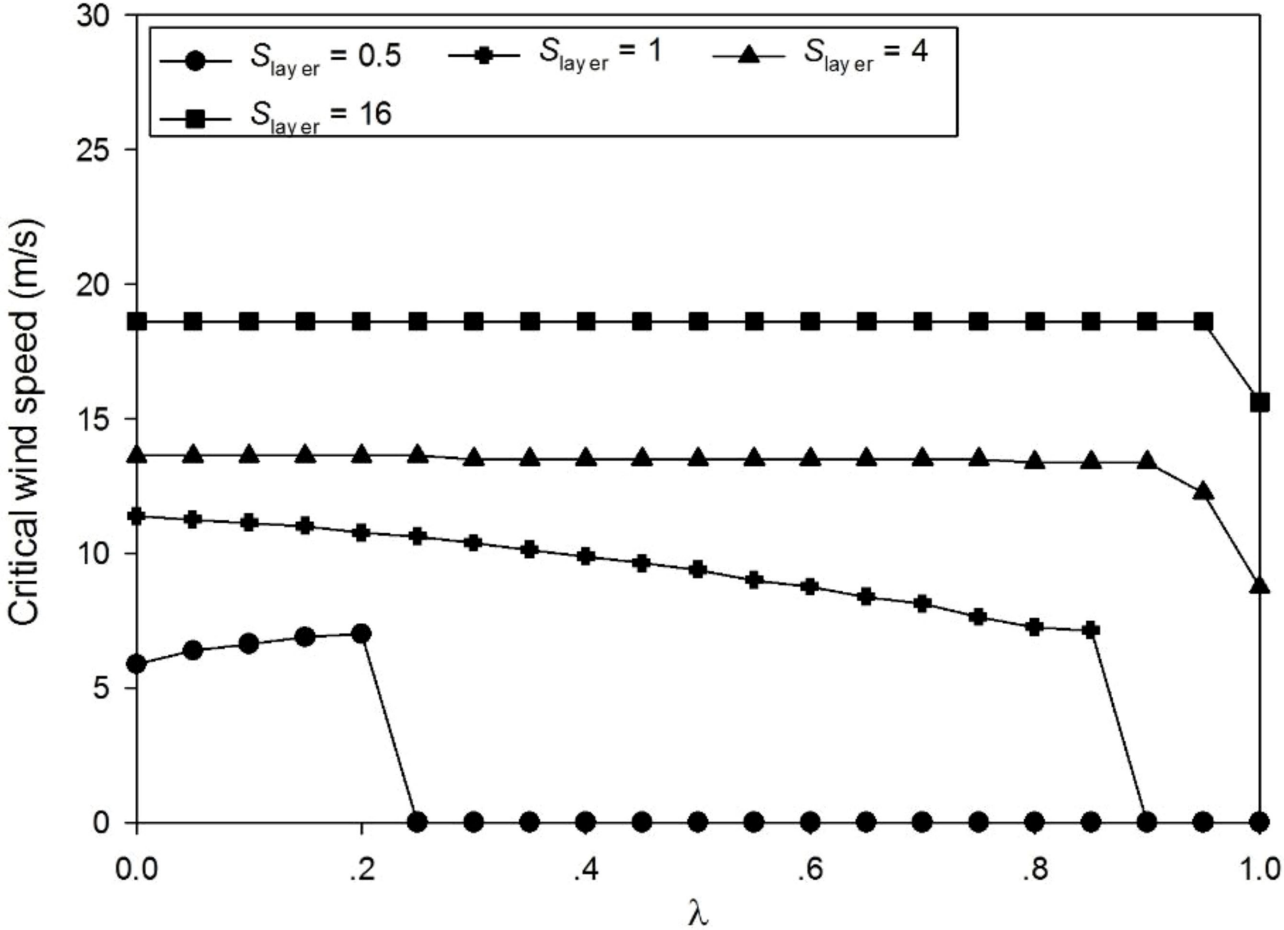
Effect of *λ* on the critical wind speed (CWS) with different *S*_layer_ values.

Differing from the conclusion that *λ* should be equal to 1 to obtain the maximal wood biomass (Qi et al., 2009), here the influence of *λ* is more complex, as shown in **Figure 8**. At a lower *S*_layer_ value, e.g., *S*_layer_ = 0.5, the trunk is thin and the diameter of the tree top is too slim to support itself. Even very small wind can cause a breakage due to self-weight. The position with the maximum value of the breakage value also moves downward along the trunk. At a higher *S*_layer_ value, e.g., *S*_layer_ = 4, the critical wind bore by the trunk becomes stronger and becomes indifferent to the *λ* value. **Table 2** shows the force and moment given *MOR* = 45 MN/m^2^. **Supplementary Figure S3** shows the 3D tree shapes under different *λ*, where the largest stress value on the trunk *Stress*_Trunk_ is marked with a black circle. Only the trunk and the primary branch structure are shown. *λ* does not change the height of the tree as it matters only the allocation of biomass for secondary growth, but it influences the stem shape and accordingly breakage. According to the numerical results, *λ* has little effect on optimization results; thus, it is not considered as a control parameter for tree reaction (set to *λ =* 0.1).

**Table 2:** The force and moment given MOR 45 MN/m^2^.

λ	*Stress_tree_*	*Stress_trunk_*	POT	H	D	*f*_x_	*f*_y_	*f*_z_	*M*_x_	*M*_y_	*M*_z_
0	16.06	3.94	1	0.00	20.37	245.53	0	-485.59	3.38	3271.61	0.20
0.1	17.44	3.12	77	1.62	20.47	218.73	0	-450.67	2.33	2623.95	-2.41
0.2	19.05	1.86	74	1.54	24.40	218.62	0	-468.49	3.43	2569.46	0.05
0.3	21.44	1.43	74	1.54	26.22	216.16	0	-477.19	2.96	2455.43	-0.05
0.4	26.26	1.14	74	1.54	27.92	213.35	0	-485.90	2.46	2340.48	-0.19
0.5	34.33	0.91	74	1.54	29.52	210.15	0	-494.60	1.90	2224.00	-0.42
0.6	49.20	0.74	74	1.54	31.04	206.52	0	-503.30	1.26	2105.41	-0.77
0.7	81.33	0.61	74	1.54	32.49	202.36	0	-512.01	0.51	1983.95	-1.38
0.8	167.62	0.50	74	1.54	33.88	197.50	0	-520.71	-0.41	1858.54	-2.53
0.9	456.40	0.42	74	1.54	35.21	191.60	0	-529.42	-1.46	1727.47	-4.91
1	140.26	0.12	39	0.61	39.01	206.62	0	-670.55	2.84	1017.44	3.84

The wind speed is 13.5 m/s. Stress_Tree_ represents the maximum stress value (MN/m^2^ or MPa) inside the whole tree. POT represents the position that bears the maximum stress value on the trunk (Stress_trunk_, MN/m^2^), which is the number of internodes counted from the base to the top. H and D represent the height (m) and diameter (cm) of POT, respectively. f_x_, f_y_, and f_z_ represent the x-, y-, and z-components of force (N) at POT, respectively. M_x_, M_y_, and M_z_ represent the x-, y-, and z-components of the moment (N·m ) at POT, respectively.

### 3.4 Plant plasticity simulation

#### 3.4.1 Pareto optimal frontier

**Figure 9:**
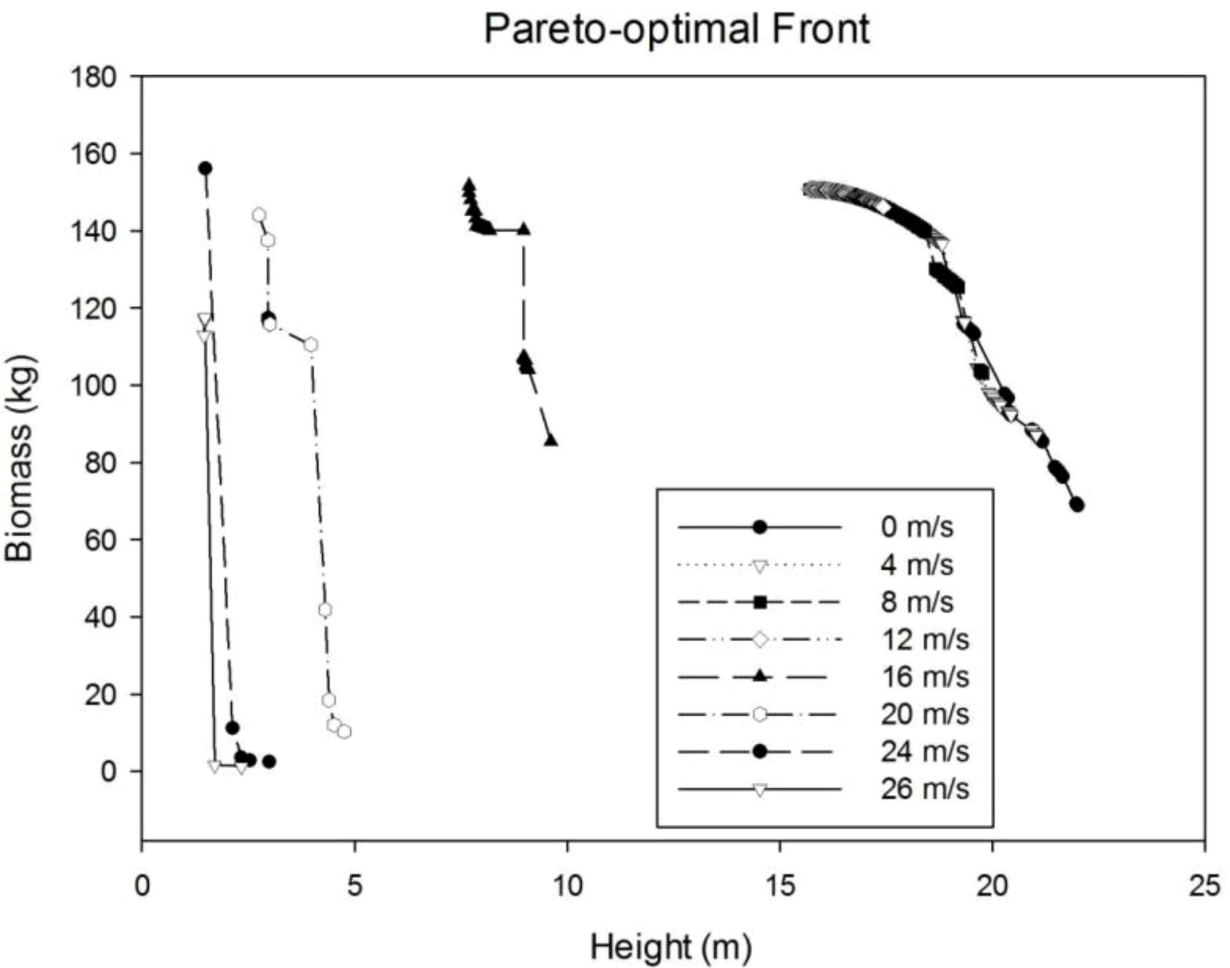
Pareto optimal front for different wind speeds.

Tree plasticity is simulated by searching the best parameter value of *S*_layer_ to maximize the tree biomass and tree height while keeping the trunk unbroken, given the wind speed. Because of multi-objective optimization, the result is no more a single point but a point set, called Pareto set or Pareto frontier (**Figure 9**). In general, when the wind speed is smaller than 16 m/s, the distribution of points in the Pareto boundary coincides with each other, which means the reaction tree is close to the relatively small wind. When the wind speed is high, as seen at the wind speed of 16 m/s, the points in the Pareto optimal front are separated from the previous ones, which means a different reaction. In both situations, according to the Pareto sets, the optimal tree can be a taller tree with smaller biomass, or a shorter tree with bigger biomass. In general, the tree height shows an obvious downward trend with wind speed. The total weight does not change significantly when the wind speed is low but has wider distribution with the increase in wind speed.

#### 3.4.2 Optimal parameter values

Each dot in the Pareto frontier corresponds to an optimal parameter, so the box diagram is used to show the dispersion degree of the optimal *S*_layer_ for each wind speed (**Supplementary Figure S4**). When the wind speed is small, the box is small, which means the parameters are more concentrated. When the wind speed increases to 16 m/s or bigger, the maximum deviation of the “outlier” from the box gradually increases, indicating that the tree tends to allocate more biomass to the secondary growth to reinforce its structure under the wind. With the increase in wind speed, the median value of each box has a gradual upward trend.

**Table 3** shows the optimal parameter values under different wind speeds according to the box diagram (**Supplementary Figure S4**) by taking representative values (the maximum, minimum, median, and the ratios of 10%, 25%, 75%, and 90%) of the box. In general, the trend is that the parameter value increased with wind speed.

**Table 3:** Optimized parameter values for different wind speeds.

Wind speed (m/s)	Minimum	Error bars below box (10th percentile)	Lower boundary of the box (25th percentile)	Media (50th percentile)	Upper boundary of the box (75th percentile)	Error bars above box (90th percentile)	Maximum
0	0.25	0.28	0.34	0.47	0.66	0.84	0.96
2	0.26	0.29	0.34	0.45	0.65	0.84	0.97
4	0.3	0.33	0.37	0.48	0.68	0.82	0.97
6	0.32	0.34	0.39	0.54	0.72	0.85	0.97
8	0.35	0.38	0.44	0.57	0.74	0.87	0.97
10	0.44	0.46	0.51	0.63	0.78	0.88	0.97
12	0.61	0.63	0.67	0.73	0.82	0.91	0.97
14	0.88	0.89	0.89	0.91	0.93	0.95	1.13
16	0.51	0.53	0.75	0.88	0.95	1	2.97
18	0.86	0.86	0.87	0.89	0.97	1.45	4.89
20	0.54	0.55	0.57	0.6	0.65	2.28	6.93
22	0.65	0.65	0.65	1.54	8.6	9.22	9.29
24	0.77	5.84	12.48	12.48	12.48	12.48	12.48
26	1.33	1.33	1.33	18.21	18.21	18.21	18.29

#### 3.4.3 Morphological result

To better analyze the morphological changes of trees reacting to different wind conditions, three points (representing three strategies) at the two ends and the middle of the Pareto frontier are selected. For each wind speed, the corresponding *S*_layer_ value of the same strategy is shown in **Table 4**. The strategy I (0% H, 100% Q) in **Table 4** represents the point corresponding to the minimal tree height and the maximal biomass in the Pareto optimal front. This point can be considered as the tree focuses on pursuing weight goals. In this case, when the wind speed is small (less than 12 m/s), the change of *S*_layer_ is not obvious, so it can be considered that it is not sensitive to the stimulation of the breeze. Strategy III (100% H, 0% Q) corresponds to the point with the largest tree height and the smallest biomass in the Pareto optimal frontier. It can be considered that in this case, trees focus on pursuing tree height but not the tree biomass. In this case, the *S*_layer_ value is taken in a small range. Strategy II (50% H, 50% Q) corresponds to a middle point in the Pareto front representing this set of optimal solutions. Its selection method is as follows: (1) average the optimal solution set corresponding to a Pareto front; (2) select an optimal solution belonging to the Pareto set that is closest to the average value to represent the whole set of optimal solutions. This strategy is a compromise between tree height and weight. The *S*_layer_ value gradually increases in strategy II.

**Table 4:** Corresponding *S*_layer_ value with different wind speeds under strategies I, II, and III.

Wind speed (m/s)	Strategy I:0% H, 100% Q			Strategy II:50% H and 50% Q			Strategy III:100% H, 0% Q		
	H_tree_ (m)	Q_stem_ (kg)	S_layer_	H_tree_ (m)	Q_stem_ (kg)	S_layer_	H_tree_ (m)	Q_stem_ (kg)	S_layer_
0	15.73	150.75	0.96	17.96	143.11	0.52	21.73	75.83	0.25
2	15.69	150.75	0.97	18.02	142.71	0.51	20.94	89.75	0.26
4	15.69	150.75	0.97	17.85	143.86	0.54	20.05	95.62	0.3
6	15.69	150.75	0.97	17.67	144.91	0.57	19.74	96.14	0.32
8	15.69	150.75	0.97	17.56	145.54	0.59	19.07	102.53	0.35
10	15.69	150.75	0.97	17.23	147.19	0.65	18.46	116.05	0.44
12	15.69	150.75	0.97	16.71	149.18	0.75	17.45	146.13	0.61
14	15.03	149.97	1.13	15.93	150.68	0.92	16.09	150.52	0.88
16	9.63	85.68	2.97	15.94	150.67	0.91	18.02	142.71	0.51
18	6.46	28.04	4.89	15.65	150.75	0.98	16.18	150.39	0.86
20	4.76	10.34	6.93	14.15	146.62	1.36	17.85	143.86	0.54
22	3.73	4.75	9.29	5.96	21.73	5.35	17.23	147.19	0.65
24	2.99	2.45	12.48	3.1	2.72	11.87	16.61	149.47	0.77
26	2.34	1.25	18.29	2.68	1.8	14.67	14.26	147.18	1.33

*H*_tree_ represents the minimal tree height, and *Q*_stem_ represents the maximal biomass in the Pareto optimal front for different strategies.

**Figure 10:**
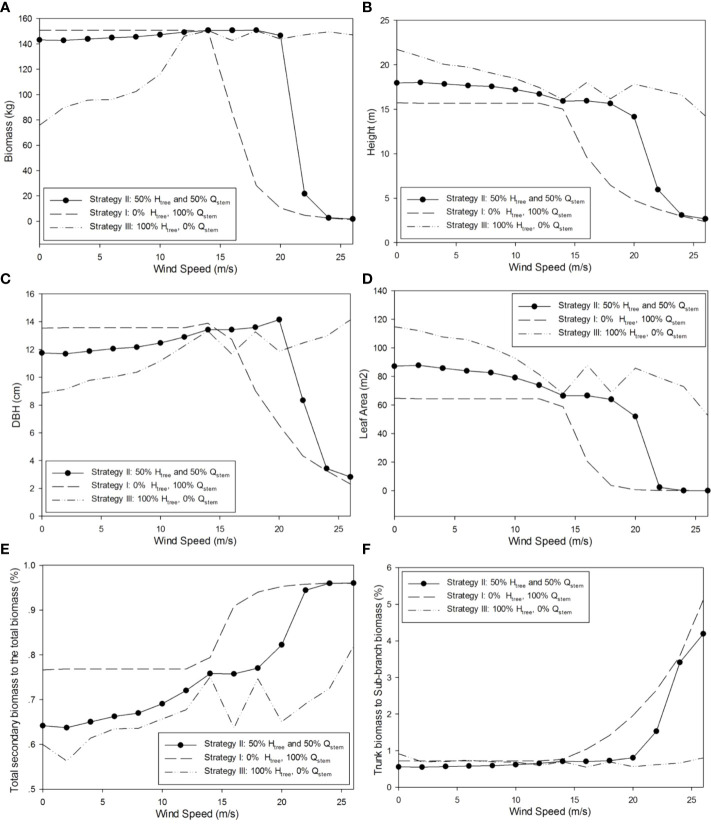
Simulated tree morphological structures reacting to wind with strategies I, II, and III. **(A)** Plant biomass (kg); **(B)** plant height (m); **(C)** trunk diameter at breast height (cm); **(D)** tree leaf area (m^2^); **(E)** biomass partitioning to secondary growth (%); and **(F)** ratio for trunk biomass to the sub-branch biomass (%).

**Figure 11:**
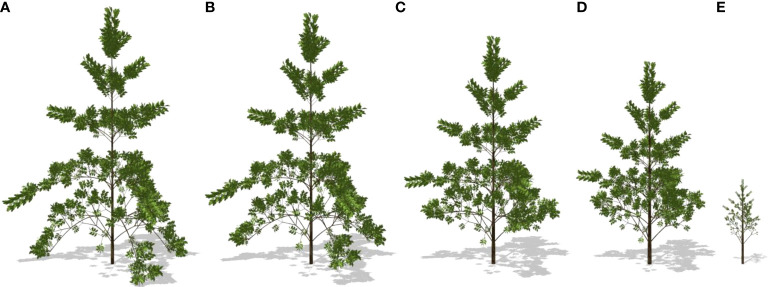
Simulated optimal tree plasticity for strategy II under different wind speeds. **(A)** 0.0 m/s; **(B)** 8 m/s; **(C)** 16 m/s; **(D)** 20 m/s **(E)** 22 m/s. Images are created under calm conditions.

Through the representative optimal result of strategy II, the plasticity in tree morphological features can be seen. With the increase in wind speed, the diameter at breast height (DBH) increased slightly with wind speed but declined significantly at a strong wind speed; the height and leaf area of the tree progressively declined, which is consistent with the experiments in the literatures (Whitehead, 1962; Telewski & Pruyn, 1998). The partitioning of biomass to the secondary growth increased, especially those for the trunk. The morphology of the tree significantly varies with the gradual acceleration of wind speed. The tree structure corresponding to strategies I, II, and III changes with the wind speed, as shown in **Figure 10**. The corresponding optimal 3D shapes of simulated poplar trees for strategy II under different wind environments are shown in **Figure 11**.

## 4 Discussion

The interest in this work was initially inspired by the phenomena of thigmomorphogenesis (Hamant, 2013), which is the plant response to biomechanical signals. Such knowledge is applied in a greenhouse by posing artificial wind to have stronger plants. Under lower light, the plant tends to be thinner (with lower biomass) yet keeps tall to catch the light (with modified allometry, i.e., the ratio between length and diameter for the internode). This is a usually unexpected phenotype as plants will be prone to breakage. Blowing the plant with wind can compensate for plants grown in a greenhouse, where light is less intensive than in an open field. An integrated modeling system with light and wind can support quantitative evaluation. However, most modeling studies on plant plasticity concentrate on the response of plants to other environmental conditions like temperature (Liu et al., 2016), light (Kang et al., 2012), or irrigation (Wu et al., 2012), without mechanical constraint. Combining the effect of both light and wind is feasible as light environment simulation has become very common in the FSPM community (Wang et al., 2012). The balance between light and wind can be simulated: to have a better light interception, the leaves need to be more dispersed, while the biomechanical constraint may require a more compact distribution of leaves. An example can be found in Eloy et al. (2017) who explored tree fitness under both light and biomechanical signals. Light competition influences plant height through both the whole tree biomass and architecture, as simulated in Cournède et al. (2008) as well as the allometries; in some cases, stronger light competition leads to higher individual plants (Chen et al., 2010). The current paper deals with trees grown in the open field where the light condition is uniform, and it is not expressed explicitly in the environmental variable *E*(*n*) (Eq. S15). Its competition level is expressed with the ground projection area *S*_p_ (Eq. S15) as described in Cournède et al. (2008). For the indoor environment as in the greenhouse, the light effect can be considered in the integrated variable *E*(*n*) as done in Fan et al. (2015). The sophisticated interplay between light and wind can be better understood by introducing a realistic light component.

Tree reaction to a biomechanical stimulus can be split into two complementary processes: (1) the reallocation of biomass within the whole plant and stem allometry changing and (2) the differentiation of wood cells (e.g., formation of reaction wood) and active biomechanical control of stem shape due to wood fiber maturation strains (Moulia et al., 2006). This paper concentrates on the first complementary mechanism by finding the parameter controlling the reallocation of biomass to secondary growth. It may be called a goal-seeking approach (Takahara & Mesarovic, 2003) in optimizing dry matter distribution with mechanical constraints: instead of simulating directly the impact of wind on plant growth, it searches for suitable growth parameters that lead to optimal biomass and height at given wind load. A similar idea of simulating plant acclimation through optimization can be found in Eloy et al. (2017), with a conceptual model. In the current work, the reaction of the tree is reflected by a single parameter *S*_layer_. Even with a constant *S*_layer_, the allocation changes because of tree organogenesis (**Figure 10E**). The results fit the situation where trees receive stable wind. When trees grow in varying wind environments, a variable *S*_layer_ is expected. Such variable value has been found on maple trees (De Reffye et al., 2017).

The numerical results show that the response of the plant to the wind is mild for a breeze. The response takes place gradually according to **Figure 10**. It is applicable for the situation where plants receive frequent wind loads for a long period. However, when the tree starts to respond to the wind load is not studied here. How plants react instantly to critical wind load that causes top stem deflection is uninvestigated. Due to the limit of experiment conditions, most experiments were done for low wind speeds. The *in silico* experiment gives simulated results under strong wind that is hard to achieve and needs further verification. For trees, through measurement and model calibration, all sink–source parameters can be estimated, which is the basis of the *in silico* experiment. In this work, the model parameters are based on both literature and previous study. The role of wind is not limited to a biomechanical stimulus; it also influences the photosynthesis process by modifying leaf boundary layer conductance, which in turn influences leaf surface CO_2_ concentration and humidity. As wind speed increases, the increasing transpiration rate can lead to a decrease in the water content of leaves (Yabuki, 2004). The simulations of such physiological processes are expected to bring more realistic results.

## 5 Conclusion

The compensating mechanism of plants in reacting to the wind environment is simulated using a system coupling a FSPM with a biomechanical model. A smaller tree (with smaller plant height and leaf area) with higher wind resistance is obtained at higher wind speed, as an emergent property of the system. Secondary growth increases to avoid trunk breakage. This work allows investigating the compensating mechanism of biomass computationally and increases the potential of FSPM for a wider application.

## Data availability statement

The original contributions presented in the study are included in the article/**Supplementary Material**. Further inquiries can be directed to the corresponding authors.

## Author contributions

MK, TF, and PDR planned and designed the research. HW performed the experiments and analyzed the data. MK, HW, JH, XW, XF and TF wrote and revised the manuscript. All authors contributed to the article and approved the submitted version.

## Funding

This work is supported by the Major S&T project (Innovation 2030) of China (2021ZD0113704), the National Natural Science Foundation of China (62076239), the CAS-NSTDA Joint Research Program (GJHZ2076), the Natural Science Startup Foundation of Chongqing Technology and Business University (2056019) and the Science and Technology Research Program of Chongqing Municipal Education Commission (KJQN201900833).

## Acknowledgments

The authors thank LIAMA for its support within the cPlant project framework.

## Conflict of interest

The authors declare that the research was conducted in the absence of any commercial or financial relationships that could be construed as a potential conflict of interest.

## Publisher’s note

All claims expressed in this article are solely those of the authors and do not necessarily represent those of their affiliated organizations, or those of the publisher, the editors and the reviewers. Any product that may be evaluated in this article, or claim that may be made by its manufacturer, is not guaranteed or endorsed by the publisher.
