# Supplementary material for: Stronger wind, smaller tree: Testing tree growth plasticity through a modeling approach
Source: Front Plant Sci. 2022 Nov 10;13:971690. doi: 10.3389/fpls.2022.971690 (PMC9686872; doi:10.3389/fpls.2022.971690)
Supplement: Supplementary file 1 [file DataSheet_1.docx]

Supplementary Material

Stronger wind, smaller tree: testing tree growth plasticity through a modelling approach

Haoyu Wang^1,2^, Jing Hua^1,2^, Mengzhen Kang^1,3*^, Xiujuan Wang^1,2*^, Xing-Rong Fan^4^, Thierry Fourcaud^5^, Philippe de Reffye^5^, Feiyue Wang^1^

*** Correspondence:** Mengzhen Kang, mengzhen.kang@ia.ac.cn

Xiujuan Wang**,** xiujuan.wang@ia.ac.cn

# Supplement A: Incremental Transfer Matrix Method (ITMM)

The detailed description of vectors in Eq. 1 is as follows.

| $\left\{ \begin{aligned} \begin{matrix} D_{k}=\left\langle u,v,w,\omega_{x},\omega_{y},\omega_{z} \right\rangle\\ S_{k}=\left\langle N_{x},V_{y},V_{z},M_{x},M_{y},M_{z} \right\rangle\end{matrix} \\ F= \left\langle f_{x},f_{y},f_{z},f_{x},f_{y},f_{z} \right\rangle\end{aligned} \right.$ | (S1) |
| --- | --- |

$D_{k}$ is vectors of generalised displacements; $S_{k} is vectors of$generalized internal forces, and *F* is the vector of linear forces, according to the beam local reference axes *x*, *y*, *z*. $f_{x}$, $f_{y}$, $f_{z}$ are loads ($N\cdot m^{-1}$); $u,v,w$ are the positive translations of cross-section centre (m); $\omega_{x},\omega_{y},\omega_{z}$ are the positive rotations of cross-section normal (radians); $N_{x}$ (normal force, N), $V_{y},V_{z}$ (shear force, N), $M_{x}$ (torsion moment, $N\cdot m$), $M_{y}$, $M_{z}$ (bending moments $N\cdot m$) are the positive internal forces.

Transformation matrix *T*_ij_ (*i*, *j* = 1 or 2) in Eq. 1 is given in the global axis by

| $T_{ij}= KT_{ij}^{local}K^{-1}$ | (S2) |
| --- | --- |

where $K=\left( \begin{matrix} P^{el} & 0 \\ 0 & P^{el} \end{matrix} \right)$ and $P^{el}=\left( \begin{matrix} x_{1} & y_{1} & z_{1} \\ x_{2} & y_{2} & z_{2} \\ x_{3} & y_{3} & z_{3} \end{matrix} \right)$

(*x*, *y*, *z*) are the local axes defined by their coordinates $x_{i}$, $y_{i}$, $z_{i}$ (*i* = 1, 2, 3) in the global axes.

| $T_{11}^{local}=\left( \begin{aligned} \begin{matrix} 1 & 0 & 0 \\ 0 & 1 & 0 \\ 0 & 0 & 1 \end{matrix} \begin{matrix} 0 & 0 & 0 \\ 0 & 0 & L \\ 0 & -L & 0 \end{matrix} \\ \begin{matrix} 0 & 0 & 0 \\ 0 & 0 & 0 \\ 0 & 0 & 0 \end{matrix} \begin{matrix} 1 & 0 & 0 \\ 0 & 1 & 0 \\ 0 & 0 & 1 \end{matrix} \end{aligned} \right)$ | (S3) |
| --- | --- |
| $T_{12}^{local}=\left( \begin{aligned} \begin{matrix} \frac{-L}{EA} & 0 & 0 \\ 0 & \frac{L^{3}}{6EI} & 0 \\ 0 & 0 & \frac{L^{3}}{6EI} \end{matrix} \begin{matrix} 0 & 0 & 0 \\ 0 & 0 & \frac{-L^{2}}{2EI} \\ 0 & \frac{L^{2}}{2EI} & 0 \end{matrix} \\ \begin{matrix} 0 & 0 & 0 \\ 0 & 0 & \frac{-L^{2}}{2EI} \\ 0 & \frac{L^{2}}{2EI} & 0 \end{matrix} \begin{matrix} \frac{-L}{GJ} & 0 & 0 \\ 0 & \frac{-L}{EI} & 0 \\ 0 & 0 & \frac{-L}{EI} \end{matrix} \end{aligned} \right)$ | (S4) |
| $T_{21}^{local}=\left( \begin{aligned} \begin{matrix} 0 & 0 & 0 \\ 0 & 0 & 0 \\ 0 & 0 & 0 \end{matrix} \begin{matrix} 0 & 0 & 0 \\ 0 & 0 & 0 \\ 0 & 0 & 0 \end{matrix} \\ \begin{matrix} 0 & 0 & 0 \\ 0 & 0 & 0 \\ 0 & 0 & 0 \end{matrix} \begin{matrix} 0 & 0 & 0 \\ 0 & 0 & 0 \\ 0 & 0 & 0 \end{matrix} \end{aligned} \right)$ | (S5) |
| $T_{22}^{local}=\left( \begin{aligned} \begin{matrix} -1 & 0 & 0 \\ 0 & -1 & 0 \\ 0 & 0 & -1 \end{matrix} \begin{matrix} 0 & 0 & 0 \\ 0 & 0 & 0 \\ 0 & 0 & 0 \end{matrix} \\ \begin{matrix} 0 & 0 & 0 \\ 0 & 0 & -L \\ 0 & L & 0 \end{matrix} \begin{matrix} -1 & 0 & 0 \\ 0 & -1 & 0 \\ 0 & 0 & -1 \end{matrix} \end{aligned} \right)$ | (S6) |

where *L* is the internode length; *E* is the Young’s modulus; $G=E/2(1+\nu)$ is the shear modulus; $\nu$ is the Poisson’s coefficient. The moments of inertia of the internode cross-section are defined with respect to the beam local reference axes *x, y, z* by:

$I_{y}= \int z^{2} dA$; $I_{z}= \int y^{2} dA$; $J= \int{(y^{2}+z}^{2}) dA$, where A is the internode cross-section area. Define $I_{y}= I_{z}=I$ and then $J=2I$.

Transformation matrix $M_{1}$ and $M_{2}$ in Eq. 1 are given in the global axis by

| $\left\{ \begin{matrix} M_{1}=\left\langle M_{11}^{3\times3} \vert M_{12}^{3\times3} \right\rangle\\ M_{2}=\left\langle M_{21}^{3\times3} \vert M_{22}^{3\times3} \right\rangle\end{matrix} \right.$ | (S7) |
| --- | --- |

where

| $M_{ij}^{3\times3}= P^{el}M_{ij}^{3\times3,local}P^{el}$ | (S8) |
| --- | --- |
| $M_{11}^{3\times3,local}=\left( \begin{matrix} \frac{-L^{2}}{2EA} & 0 & 0 \\ 0 & \frac{L^{4}}{24EI} & 0 \\ 0 & 0 & \frac{L^{4}}{24EI} \end{matrix} \right)$ | (S9) |
| $M_{12}^{3\times3,local}=\left( \begin{matrix} 0 & 0 & 0 \\ 0 & 0 & \frac{-L^{3}}{6EI} \\ 0 & \frac{L^{3}}{6EI} & 0 \end{matrix} \right)$ | (S10) |
| $M_{21}^{3\times3,local}=\left( \begin{matrix} -L & 0 & 0 \\ 0 & -L & 0 \\ 0 & 0 & -L \end{matrix} \right)$ | (S11) |
| $M_{22}^{3\times3,local}=\left( \begin{matrix} 0 & 0 & 0 \\ 0 & 0 & \frac{{-L}^{2}}{2} \\ 0 & \frac{L^{2}}{2} & 0 \end{matrix} \right)$ | (S12) |

| $M_{a}=K{[\varepsilon\cdot L, K_{z}\cdot\frac{L^{2}}{2}, 0, 0, 0, K_{z}\cdot L]}^{T}$ | (S13) |
| --- | --- |

*M*a denotes the maturation strains (MS) at the periphery of stems, associated with the formation of reaction wood.

where

| $\left\{ \begin{aligned} \varepsilon=\frac{E_{n}\cdot A_{n}}{{(E\cdot A)}_{n}}\left( a+\frac{\xi}{2}\left( b-a \right) \right) \\ K_{z}=-\frac{\pi\xi E_{n}}{6\cdot{(E\cdot I)}_{n}}\left( b-a \right)\left( R_{n}^{3}-R_{n-1}^{3} \right)\cos\psi\end{aligned} \right.$ | (S14) |
| --- | --- |

where $A_{n}$ is the cross-section area of the peripheral ring *n*. $\xi$ is a parameter defining the strategy of straightening up. Basically, $\xi$ =1 if reaction, $\xi$ = 0 else, but intermediate values could be used to modulate the process. $R_{n}$ denote the external radius of the peripheral ring *n*. *a* and *b* define the extreme values of maturation strains, for normal wood and reaction wood respectively. Concerning the negative-gravitropism of coniferous trees, longitudinal elongation of compression wood cells is represented by positive values of *b* reached at the lower part of the beam, i.e. at $\psi=$0. However, tension wood shrinkage is defined by negative values of *b* reached at $\psi=\pi$ for broadleaf trees. *a* and *b* can be estimated by measuring the longitudinal residual maturation strains on living trees using different techniques.

# Supplement B: GreenLab Model

Here we recall the key functions dedicated to trees. Generally, GreenLab consists of two basic processes: development (organogenesis) and growth. The organogenesis is simulated with an automaton, which gives the topological structure and organs inside the tree structure at each age. In this study, the organogenesis model of the poplar tree (Yang et al., 2011) is taken. Organs of the same type are distinguished by physiological age (PA), a botanic variable characterizing the morphological differentiation of organs (Letort et al., 2008). The growth of an organ is regulated by the source-sink balance (the ratio of biomass production *Q* and plant demand *D*, *Q*/*D*). The primary growth of organs (leaves, internodes and fruits) is based on the common pool hypothesis, while the secondary growth of the stem is dependent on the number of leaves 'seen' by each part of the stem.

## Biomass production

At each growth cycle (GC) *n*, biomass production *Q*(*n*) is described below, as shown in Eq. S15.

| $\left\{ \begin{matrix} Q\left( n \right)=E(n)\mu S_{p}\left( 1-\exp\left( -k\frac{S(n)}{S_{p}} \right) \right) \\ Q\left( 0 \right)=Q_{seed} \end{matrix} \right.$ | (S15) |
| --- | --- |

where *E*(*n*) is a variable representing the potential evapotranspiration at GC *n*; *µ* denotes the water-use efficiency; *S*_p_ is the total ground projection area of the plant crown; *k* is a light extinction coefficient, which is used to quantify the attenuation process of light penetrating the canopy; *S*(*n*) is the whole green leaf surface area at GC *n*; *Q*_seed_ is the initial biomass of the plant. The ratio *S*(*n*)/*S*_p_ can be considered as a local leaf area index (LAI), which is an important value to evaluate light interception (Cournède et al., 2008; Letort et al., 2008). It influences individual biomass production and contributes to light competition in stands. Note that *Q*(*n*) and *S*(*n*) are state variables that change during plant growth, and can be influenced by wind speed.

## Biomass demand

All growing organs are sinks among which the biomass is distributed according to their sink values. For biomass partition inside the tree, two aspects are considered: primary and secondary growth. The primary growth corresponds to the emergence and the expansion of new shoots. The secondary growth corresponds to the radial increment of existing phytomers, with new layers of wood formed around the stem periphery. The biomass demand of plants for the primary growth at GC *n*, denoted by $D_{\mathrm{pri}}^{o}(n)$, is given by:

| $D_{\mathrm{pri}}^{o}\left( n \right)=\sum_{o} \sum_{p=1}^{P_{m}} P_{p}^{o}N_{p}^{o}(n)$ | (S16) |
| --- | --- |

where $P_{p}^{o}$ is the sink strength of organ *o* with the PA *p* (*P*_m_ being the maximum PA); *o* representing different organs (b: blade, i: internode, r: ring); the leaf of the trunk being the reference, i.e., $P_{1}^{b}$=1; for year-based tree model, each GC corresponds to one year, so expansion is immediate. $N_{p}^{o}(n)$ is the number of new organs created at GC *n*, produced by the organogenesis model.

Total plant demand *D*(*n*) is the sum of demand for the primary growth (Eq. S16) and those for the secondary growth (Eq. 2), as described in Eq. S17:

| $D\left( n \right)=D_{\sec}^{\mathrm{layer}}\left( n \right)+D_{\mathrm{pri}}^{o}\left( n \right)$ | (S17) |
| --- | --- |

## Size of organ

The length and diameter of individual growth unit are necessary for the biomechanical model. As mentioned, while the length of the internode is given by primary growth, the diameter is contributed by both primary and secondary growth.

The volume of internode born from primary growth is given by:

| $v_{\mathrm{pri},p}^{i}\left( n \right)=q_{\mathrm{pri},p}^{i}\left( n \right)/\rho_{p}^{\mathrm{pith}}$ | (S18) |
| --- | --- |

where$\rho_{p}^{\mathrm{pith}}$ is the density of pith created at plant age *n*. With allometric law (Yan et al., 2004), the length of internode in Eq. S19 is:

| $l_{p}(n)=\sqrt{\beta\cdot{v_{\mathrm{pri},p}^{i}\left( n \right)}^{\frac{1+\alpha}{2}}}$ | (S19) |
| --- | --- |

where $\alpha$ and $\beta$ are allometric parameters.

The section area of pith is:

| $s_{p}^{\mathrm{pith}}\left( n \right)=\frac{q_{\mathrm{pri},p}^{i}\left( n \right)}{\rho_{p}^{\mathrm{pith}}\cdot l_{p}(n)}$ | (S20) |
| --- | --- |

## Biomass allocation

At each GC, total biomass *Q*(*n*) (Eq. S15) is allocated to two compartments: $Q_{\mathrm{pri}}^{o}(n)$ for the primary growth and $Q_{\sec}^{\mathrm{layer}}(n)$ for the secondary growth of organ *o* at GC *n*. The biomass partitioned to each organ for the primary growth is proportional to the source-sink ratio (*Q*/*D*): at plant age GC *n*, the amount of biomass allocated to a new-born organ *o* with the PA *p* is calculated as follows:

| $q_{\mathrm{pri},p}^{o}\left( n \right)=P_{p}^{o}\frac{Q(n)}{D(n)}$ | (S21) |
| --- | --- |

Pressler rule (Assmann, 1970) is introduced to compute the partition of $Q_{\sec}^{\mathrm{layer}}\left( n \right)$ to each phytomer. This rule assumes that the area increment on any part of the tree stem is proportional to foliage capacity in its upper part. According to Letort et al. (2008), in GreenLab, a flexible way is taken to integrate two partitioning modes using a coefficient *λ*. One model is uniform allocation (*D*_1_) along a stem, and the other is based on the Pressler rule (*D*_2_) (Letort et al., 2008; Qi et al., 2009), as in Eq. S22:

| $\left\{ \begin{matrix} D_{1}\left( n \right)=\sum_{p=1}^{P_{m}} \sum_{j=1}^{n} N_{p}^{i}\left( j \right)\cdot l_{p}(j)\cdot P_{p}^{r} \\ D_{2}\left( n \right)=\sum_{p=1}^{P_{m}} \sum_{j=1}^{n} N_{p}^{i}\left( j \right)\cdot s_{p}^{a}\left( j,n \right)\cdot l_{p}(j)\cdot P_{p}^{r} \end{matrix} \right.$ | (S22) |
| --- | --- |

where $N_{p}^{i}\left( j \right)$ is the number of internodes of PA *p* created at GC *j*; $l_{p}(j)$ is the length of the corresponding internode; $s_{p}^{a}\left( j,n \right)$ is the total area of living leaves above that phytomer at GC *n*, summed from individual leaf area. $P_{p}^{r}$ is the relative partitioning sink strength for secondary growth of internodes of PA *p*, assuming that branches of each physiological age share the same sink strength.

For leaf, it is simpler as there are no components for secondary growth. The area of an individual leaf of PA *p* created at GC *n* is:

| $s_{p}^{b}\left( n \right)=q_{pri,p}^{b}\left( n \right)/e$ | (S23) |
| --- | --- |

where *e* is the specific leaf weight.


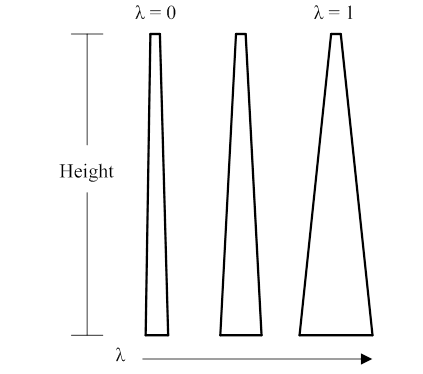


**Supplementary Figure S1.** Effect of the *λ* value on the morphology of the trunk. The trunk becomes more tapered as *λ* tends to 1


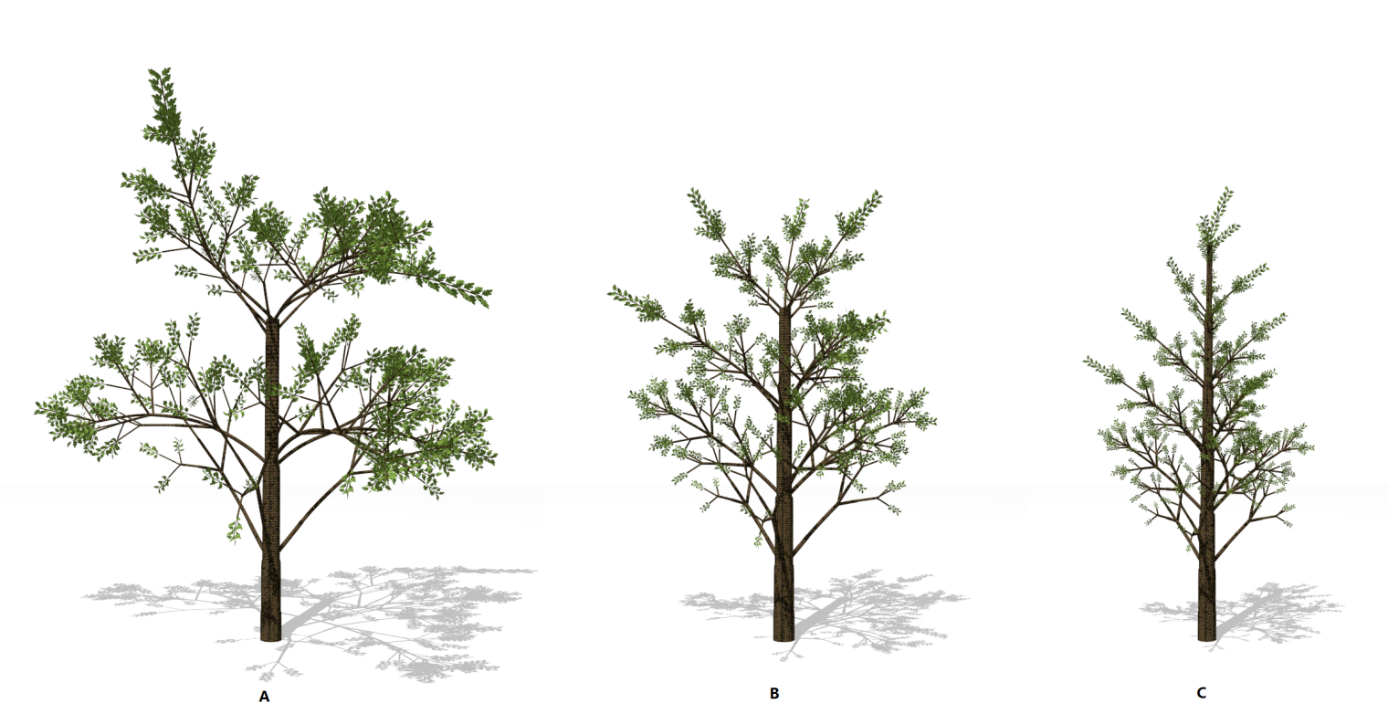


**Supplementary Figure S2.** 3D tree shape at different *S*_layer_ values. *λ* = 0.1, wind speed is 18 m/s. (a) *S*_layer_ = 2; (b) *S*_layer_ = 5; (c) *S*_layer_ = 8


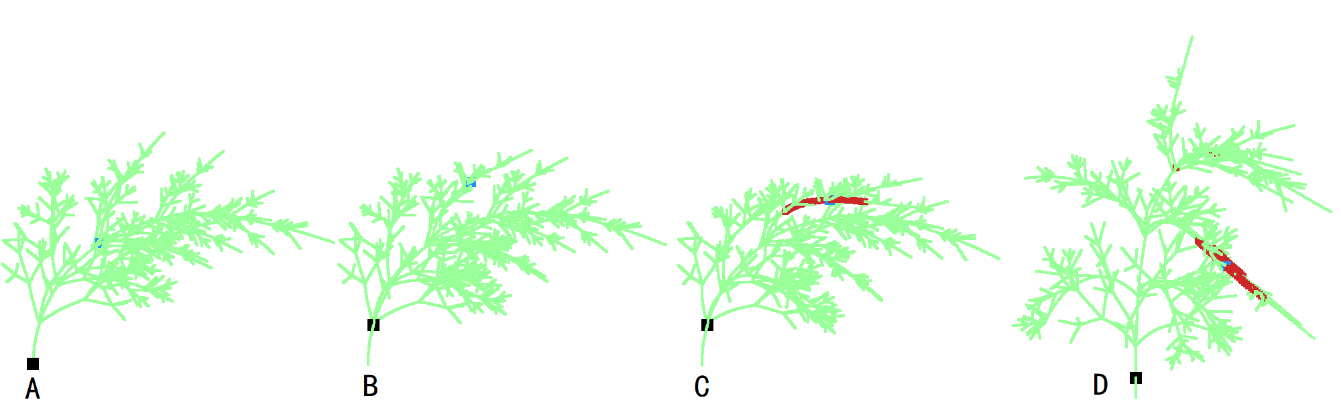


**Supplementary Figure S3.** Effect of *λ* values on the 3D form of the tree. The red points indicate the point with the maximal stress value on the trunk. (A) *λ* = 0.0, (B) *λ* = 0.4, (C) *λ* = 0.7, (D) *λ* = 1.0. The black square represents the positions of breakage ${Stress}_{Trunk}$_,_ blue represents the positions of the breakage ${Stress}_{Tree}$_,_ and red represents the positions of the breakage value above the *MOR*. The *S*_layer_ value is 4 and the wind speed is 13.5m/s


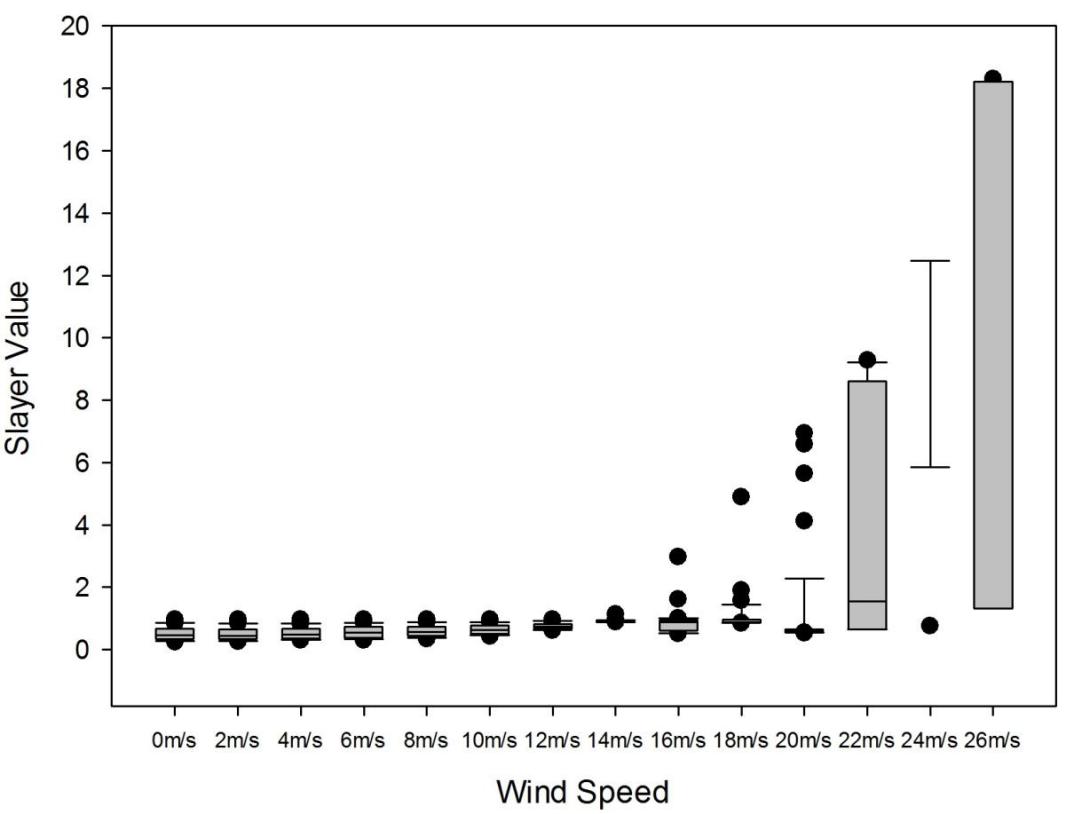


**Supplementary Figure S4.** Box diagram of optimization results with different wind speeds.

**References**

Assmann E. (1970). *The principles of forest yield study. Studies in the organic production, structure, increment and yield of forest stands*: Pergamon, Oxford.

Cournède P-H, Mathieu A, Houllier F, Barthélémy DD, de Reffye P. (2008). Computing competition for light in the GREENLAB model of plant growth: a contribution to the study of the effects of density on resource acquisition and architectural development. *Annals of Botany* 101, 1207-1219.

Letort V, Cournède PH, Mathieu A, de Reffye P, Constant T. (2008). Parametric identification of a functional-structural tree growth model and application to beech trees Fagus sylvatica. *Funct. Plant Biol.* 35, 951-963.

Qi R, Letort V, Kang M, Cournede P-H, de Reffye P, Fourcaud T. (2009). Application of the GreenLab model to simulate and optimize wood production and tree stability: a theoretical study. *Silva Fennica* 43, 465-487.

Yang G, Cao W, Liu D, Kang M, Letort V, Xing M, Huang X. (2011). Growth and development simulation based on functional-structural model GreenLab for poplar (Salicaceae). *Afr. J. Agric. Res.* 6, 3071-3077.
